# Are physiological and behavioural responses to stressors displayed concordantly by wild urban rodents?

**DOI:** 10.1007/s00114-020-01716-8

**Published:** 2021-01-07

**Authors:** Loren L. Fardell, Miguel A. Bedoya-Pérez, Christopher R. Dickman, Mathew S. Crowther, Chris R. Pavey, Edward J. Narayan

**Affiliations:** 1 School of Life and Environmental Sciences, The University of Sydney, Sydney, New South Wales 2006 Australia; 2 School of Psychology, The University of Sydney, Sydney, New South Wales 2006 Australia; 3 Brain and Mind Centre, The University of Sydney, Sydney, New South Wales 2006 Australia; 4 CSIRO, Land and Water, PMB 44, Winnellie, Northern Territory 0822 Australia; 5 School of Agriculture and Food Sciences, The University of Queensland, Brisbane, Queensland Australia

**Keywords:** Stress response, Predator avoidance, Corticosterone, Faecal glucocorticoid, Non-invasive monitoring, Wildlife

## Abstract

Understanding wild animal responses to stressors underpins effective wildlife management. In order for responses to stressors to be correctly interpreted, it is critical that measurements are taken on wild animals using minimally invasive techniques. Studies investigating wild animal responses to stressors often measure either a single physiological or behavioural variable, but whether such responses are comparable and concordant remains uncertain. We investigated this question in a pilot study that measured responses of wild-caught urban brown and black rats (*Rattus norvegicus*, *Rattus rattus*) to fur-based olfactory cues from a predator, the domestic cat (*Felis catus*); a novel herbivore, the koala (*Phascolarctos cinereus*); and a familiar herbivore and competitor, the common brushtail possum (*Trichosurus vulpecula*). Physiological responses, measured by assaying faecal glucocorticoid metabolites, were compared to behavioural responses observed via video recordings. We found that physiological and behavioural responses to stressors were expressed concordantly. There was no sizeable physiological response observed, and the behavioural response when considered across the night was negligible. However, the behavioural response to the predator and competitor cues changed across the observation period, with activity increasing with increasing hours of exposure. Our results indicate that responses of wild rodents to cues are nuanced, with stress responses modulated by behaviour changes that vary over time according to the severity of the perceived threat as animals gather further information. If the physiological response alone had been assessed, this moderated response may not have been evident, and in terms of wildlife management, vital information would have been lost.

**Supplementary Information:**

The online version contains supplementary material available at 10.1007/s00114-020-01716-8.

## Introduction

Anthropogenic disturbances are increasing the incidence of novel interactions between people, wildlife, and the environment, creating an imperative for conservation biologists to better understand and manage wild animal stress responses (Clinchy et al. 2016; Carthey and Blumstein 2018; Otto 2018; Fardell et al. 2020). Responses to a stressor depend on the perceived threat and its interpretation (Boonstra 2013) and may be the product of adaptation or plasticity and habituation. Such responses will benefit individual survival if they reduce physiological stress or remove the threat, for example by moving away or entering torpor (e.g. at signs of extreme weather or an approaching fire: Nowack et al. 2017; Abernathy et al. 2019; Nimmo et al. 2019) or altering behaviours (e.g. in response to a predator: Laundré et al. 2001). Responses to a chronic stressor, however, can have negative ecosystem-level effects, if, for example, herbivores select different plant species to forage and influence ecosystem productivity and dynamics (e.g. Hawlena and Schmitz 2010). Being able to accurately measure and interpret the full scope of wild animals’ responses to stressors can aid our understanding of potential threats and guide management actions to reduce them (Cockrem 2005; Wikelski and Cooke 2006; Busch and Hayward 2009; Dantzer et al. 2014).

In vertebrates, physiological stress is commonly defined as an adaptive response to a stressor, or stimulus that is aversive to the individual (Selye 1936). The function is to restore internal homeostasis (Cannon 1932) within the context of allostasis—the maintenance of internal consistency through natural changes that occur with season, age, and sex (McEwen and Wingfield 2003). Vertebrates can respond physiologically to a stressor through changes to the hypothalamic-pituitary-adrenal (HPA) axis, which causes changes in the secretion of adrenal glucocorticoids (Wingfield and Ramenofsky 1999)*.* Accordingly, one way to measure physiological stress is via glucocorticoid hormone levels, using minimally invasive techniques to assay fur, feathers, scats, or urine (Sheriff et al. 2011; Cook 2012; Palme 2019). Glucocorticoid responses to stressors, however, often show mixed results within and across species, in part due to individual and population response variations (e.g. Koolhaas et al. 2010; Cockrem 2013), but perhaps also due to erroneous interpretations or limitations if only one index of stress is used (Davis et al. 2008; Busch and Hayward 2009; Cooke et al. 2014). Using an integrative approach to simultaneously identify multiple responses to a stressor can clarify results and help illuminate the ability of animals to cope with stressors in their natural environment (Cooke et al. 2014).

Observation of behavioural responses via remote cameras is being increasingly utilised in wildlife conservation (Caravaggi et al. 2017). Because behavioural responses to stressors have been extensively researched, they can be readily identified. Behavioural “coping” responses to a stressor range from active/proactive—originally defined as fight-flight (Cannon 1915), which is characterised by aggression or territory control (Koolhaas et al. 1999)—to passive/reactive—originally defined as conservation-withdrawal (Engel and Schmale 1972), which is characterised by immobility and low aggression (Koolhaas et al. 1999). A behavioural response may be deemed a “coping” mechanism when it is repeatedly observed in response to a recurring or chronic stressor, and can be an adaptive response shaped by evolution under sometimes aversive conditions (Wechsler 1995; Koolhaas et al. 2010). A fundamental expectation is that the physiological HPA axis response should result in a predictable behavioural response, be it short term to an acute stressor or long term to a chronic stressor (e.g. sharpened cognition, decreased feeding, suppressed breeding behaviour; Sapolsky et al. 2000).

Despite expectations, the alignment between behavioural and physiological responses to stressors is not always observed, especially in studies of wild populations (e.g. Mappes et al. 1998; Bramley et al. 2000; Jonsson et al. 2000; Carthey and Banks 2018; Stryjek et al. 2018; Mazza et al. 2019; Westrick et al. 2019). This is in part because, despite there being a many studies on the responses of wild animals to stressors, much of the theory on which the expectations for animal responses to stressors arise is based on studies of laboratory-raised animals (Boonstra, 2013; Fendt et al. 2020). Wild animals are likely to respond differently to stressors than their laboratory-raised counterparts, because the survival likelihood of wild prey individuals may be improved by their ability to perceive stressors like high predation risk and mount adaptive behavioural responses accordingly (Bókony et al. 2009).

The development of an effective predator response (see Lima and Dill 1990) that does not overextend an energy budget has clear survival benefits (e.g. Dickman 1992). It follows then that fear of predators can be a driver of evolutionary adaptations (Tooby and Cosmides 1990) that lead to moderated responses. For example, the threat-sensitive predator avoidance hypothesis postulates that prey evaluate predation risk via cues and calibrate a response that minimises the costs of responding—such as missed foraging opportunities—if the risk is low (Helfman 1989). Such nuanced responses to stressors in wildlife make it unreliable to extrapolate theory from laboratory-raised animals to their wild counterparts, in particular to expect concordance between behavioural and physiological responses of wild animals to stress (Boonstra 2013).

Wild animals may modulate their stress response according to many factors, including life-history stage, sex, season, location, habitat, previous experience, stressor type (Johnstone et al. 2012; Boonstra 2013), and food availability (i.e. the predation-sensitive food hypothesis: Sinclair and Arcese 1995). Responses to a stressor may be managed by either physiological or behavioural modulation depending on the context (Johnstone et al. 2012). As not all of these factors can be readily controlled or measured with minimally invasive methods in situ, especially those that depend on animals’ perceptions and experiences (Johnstone et al. 2012; Boonstra 2013), single focused measures of either physiological (e.g. glucocorticoid) or behavioural observations may be insufficient indicators of whether responses to a stressor are occurring.

Here, we test whether wild small mammal physiological and behavioural responses to stressors are expressed concordantly, and whether they are modulated. We conducted a pilot study using minimally invasive techniques that are appropriate for observing wild populations in situ. We observed the physiological response by assaying faecal glucocorticoid metabolites, and compared this to behavioural responses measured through remote video recording. As this was a pilot study, a small number of urban wild-caught rats (*n* = 8) were placed in outdoor arenas and their responses to the stressor of predator presence were observed. Domestic cat (*Felis catus*) fur was used as the stressor, as cats are major predators of urban rodents and influence their movement and activity (Parsons et al. 2018). We, therefore, posit that wild urban rodents will recognise cat fur as a stressor and initiate a response that is modulated and possibly not expressed concordantly in physiological and behavioural changes, but will be a more subdued than the responses observed in laboratory-raised rodents.

## Materials and methods

### Trapping and containment

Eight individual rats (six *Rattus norvegicus* and two *R*. *rattus*) were captured by cage-trapping (Tomahawk 602, 40.6 × 12.7 × 12.7 cm, Tomahawk Live Trap LLC) using a mixture of peanut butter, rolled oats, and honey as bait. Trapping occurred in September and October 2018 on the University of Sydney campus in Camperdown, New South Wales (NSW), Australia. Traps were opened before sunset and checked around sunrise each morning. After capture, rats were transported to the Fauna Park at Macquarie University, Macquarie Park, NSW, for housing and testing in outdoor enclosures. Both *R. norvegicus* and *R*. *rattus* were introduced at the time of European settlement and are widespread in Australia’s urban and natural habitats (Cronin 2000).

Prior to the commencement of the experiment, each rat was weighed, and sex and breeding condition were determined (Krinke et al. 2000; Jackson 2012). The rats were housed individually in enclosures made from aviary wire mesh (12 mm × 12 mm openings and 0.7 mm gauge) that measured 1.8 m (W) by 1.8 m (L) and 0.6 m (H). The four sides were covered with opaque plastic, and the top was uncovered. The enclosures were located outdoors, within a 50-m^2^ predator-proof aviary, open to the elements. Shade cloth was secured around (> 1 m from the enclosures’ walls) and above (> 2 m above the enclosure top) to offer protection from heat and avian predation. A fully concealed nest hide box, made from timber (23 cm L × 30 cm W × 40 cm H) with a single circular entrance (7 cm diameter), was secured to one side of each enclosure. An open-ended hut, made from transparent red perplex (30.5 cm L × 28 cm W × 25 cm H), was secured at the opposite end of the enclosure from the nest hide box. A food hopper made from open mesh wire was secured to the underside of the hut to offer protection from the weather. The food hopper mesh wire opening was smaller than the diameter of the food pellets offered (standard laboratory rodent feed), thus forcing the rats to feed by chewing at the pellets through the mesh opening, and preventing them from harvesting and storing any food items during the trials. Four water bottles were secured to the walls of the enclosure at the sides of the hide box. Food and water were accessible ad libitum during the study. The enclosures were lined with wood shavings to a depth of ~ 5 cm. Infrared cameras were set above each enclosure and connected to a computer where an ANY-maze Video Tracking System (Stoelting Co. 1999–2019) was used to record animal movements over the nocturnal activity period. Two automatic infrared spotlights (Long Range Infrared Spotlight, Jaycar, Australia) were fitted above each enclosure to supplement lighting for filming purposes. The infrared spotlights were automatically activated when environmental illumination was lower than 1 lx.

### Response to predator cues as a stressor—experimental design

Experiments were run over two periods, due to the limited number of cages available. During each period, four different individuals (three *R*. *norvegicus* and one *R*. *rattus* each time, *n* = 8) were tested. Each experiment ran for 18 nights (September 28 to October 15, and October 16 to November 2, 2018). All rats were male, apart from one female *R*. *rattus* that was used in the second experiment. Rats were left undisturbed in their enclosures for five nights to acclimatise, as confirmed by video observations of frequent exploratory, feeding, and drinking behaviours. On the sixth night, experimental manipulations of olfactory cues began.

We used three different odours: domestic cat fur, koala (*Phascolarctos cinereus*) fur, and common brushtail possum (*Trichosurus vulpecula*) fur. Domestic cat fur was obtained from groomers and veterinary clinics in Sydney. Domestic cats are frequently sighted across the University of Sydney campus, in the locations where the rats were captured, and likely exert some level of predation pressure on the rats. Koala fur was collected from koalas on the Liverpool Plains (31.48° S, 150.68° E, NSW) as part of another research project by M. Crowther. Common brushtail possum fur was collected from animals trapped on the University of Sydney campus. Koala and common brushtail possum fur were used as non-predatory pungent controls, as a novel and a familiar herbivore (and competitor), respectively. Rats captured on the University of Sydney campus were not expected to have had any contact with koalas, as they are not present on the campus, whereas common brushtail possums often scavenge in rubbish bins on the campus in proximity to the rats and act as potential competitors.

Rodents can discriminate the odour of individual cats (Staples et al. 2008). To avoid potential habituation to a treatment cue, a new mix of fur from several different individuals for the treatment species was used for each single night exposure period. Fur from each of the three species was stored individually at − 4 to − 20 °C when not in use. About 3 g of fur was used for each olfactory treatment and was presented in a tea strainer (6 cm diameter) hung next to the food hopper in the hut. To mimic an animal’s body heat, the fur was wrapped around a HotHands^®^ hand warmer (Bowen et al. 2013) that had been previously observed to maintain a constant heat of 40 °C for 10 h. Each treatment was installed in the hour before sunset and removed within 2 h of the following sunrise. Video recordings of each enclosure started at sunset and ran for a minimum of 10 h, until sunrise. Sunset and sunrise times were determined through the open-source sun distance calculator application (SunCalc.net), based on the location of the enclosures.

**Fig. 1:**
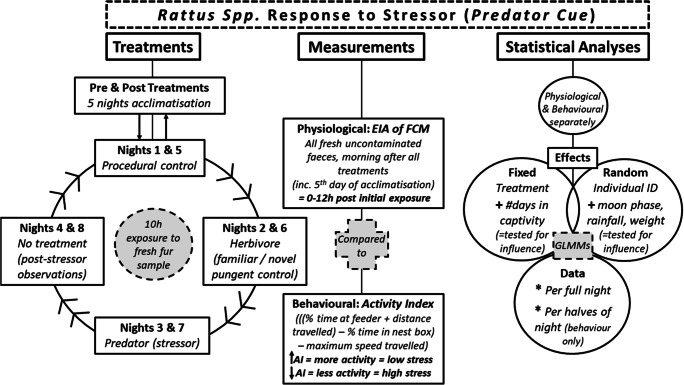
Summary diagram of the treatment exposure order, measurements taken, and the statistical analyses conducted, to assess whether the physiological and behavioural stress responses of wild-captured rats (*Rattus norvegicus* and *R*. *rattus*) to a stressor are expressed concordantly and if there is any form of stress modulation. The stressor used was domestic cat (*Felis catus*) fur (two exposures on separate nights), and the pungent controls were a single exposure to the novel herbivore koala (*Phascolarctos cinereus*), or the known competitor, the common brushtail possum (*Trichosurus vulpecula*) (a single exposure to each). The procedural control was autoclaved cat fur, as this elicits no response from rats (*pers obs.* Bedoya-Pérez 2018). Each treatment night, a fresh mix of fur was secured by the food source in the hour before sunset and removed within 2 h of the following sunrise (~ 10-h exposure time). Responses to each treatment exposure were pooled per treatment type for statistical analyses. The final models are given in the tables of model output

Olfactory treatments were presented as one per night, after the five-night acclimatisation period, in two runs that allowed for repeat exposure to the treatments. For each run, the order was as follows (Fig. 1): (1) first night, a procedural control comprising autoclaved domestic cat fur that had been shown previously to elicit no response from rats (*pers obs.* Bedoya-Pérez 2018); (2) second night, familiar herbivore pungent control (common brushtail possum fur); (3) third night, predator odour (domestic cat fur); (4) fourth night, post-predator observation (i.e. no treatment was presented), and run 2: (5) fifth night, a procedural control of autoclaved domestic cat fur; (6) sixth night, novel herbivore pungent control (koala fur); (7) seventh night, predator odour (domestic cat fur); (8) eighth night, post-predator observation with no treatment presented; (9) nights 9–13, post-treatment acclimatisation (i.e. no treatment was presented). At the end of each experiment, rats were euthanised by intraperitoneal injection of sodium pentobarbitone (Lethabarb Euthanasia Injection, Virbac (Australia, Pty Ltd)), and a blood sample was taken to test for toxoplasmosis using a commercial modified agglutination test (MAT) kit (Toxo-Screen DA, bioMérieux, France). Toxoplasmosis tests were conducted as rats infected with *Toxoplasma gondii* usually show an opposite anti-predator response towards domestic cats (Berdoy et al. 2000; Vyas et al. 2007; Hari Dass and Vyas 2014).

### Physiological response monitoring

Faecal corticosterone metabolite measurements in rodent species have previously been validated, using enzyme immunoassays (EIA) (Lepschy et al. 2007; Siswanto et al. 2008; Abelson et al. 2009; Thanos et al. 2009). As wild animals are unaccustomed to capture and human handling, faecal samples were collected the morning after capture of each rat to be used as a biological stressor control, an elevated level to compare rats’ subsequent responses. To accommodate the possibility of an acute response to the capture process impairing the ability of the wild animals to show a measurable physiological response to a stressor the day after (Dickens et al. 2009), a 5-day acclimatisation period was used before treatments were initiated. Similarly, a 24-h non-treatment period was used following exposure to the domestic cat fur (stressor) treatment. Fresh faecal samples were collected from each cage the morning of the fifth day of both pre- and post-acclimatisation to use as comparative baseline measures under the experimental conditions. Fresh faecal samples were also collected the morning after every treatment, including the non-treatment post-predator period. All faeces that were determined to be fresh, based on their moist appearance and placement in areas that had been raked clean before the treatment was administered, were collected. Samples deemed to be contaminated with urine, based on the damp appearance of the faeces and the surrounding wood shaving substrate, were discarded to avoid possible cross-contamination or degradation.

Faecal glucocorticoid metabolite concentrations increase in rat species over periods of 6–24 h post-intervention (Lepschy et al. 2007; Abelson et al. 2009). However, a peak response at around 8 h post-intervention has been most consistently observed (Siswanto et al. 2008; Thanos et al. 2009). To confirm that responses were not being expressed at the peak level 24 h after exposure, the 1-day post-cat non-treatment was put in place. Being nocturnally active, the wild-captured rats did not exit the hide box for extended periods during daylight hours, so fresh faecal samples could only be consistently collected the following morning after being deposited 0–12 h after the initial exposure. The faecal corticosterone metabolite (FCM) levels observed were, therefore, compared across the same reactive period that most likely included the peak response. To assure that exposures to environmental conditions and microbial action were minimised, faeces were collected within 2 h of sunrise, and only then if they were dry from rain, under the protection of the feeding hut (Millspaugh and Warhburn 2003, 2004; Möstl et al. 2005; Barja et al. 2012).

Samples were collected in micro-centrifuge tubes and immediately frozen at − 20 °C until analysis. Faecal samples were transported to Western Sydney University to be assessed for FCM levels using EIA. They were thawed in the fridge until they become soft, and corticosterone metabolites were extracted by suspension in 90% ethanol. Following Palme et al. (2013), each wet faecal pellet was weighed, and a 10:1 amount of 90% ethanol was added depending upon the mass (e.g. 0.125 g in 1.25 mL). Samples were then homogenised in the ethanol solution by pulverising with a spatula, vortexing for a minute, and centrifuging for 10 min at 10,000 rpm. The extracts were assayed for FCM using EIA with a polyclonal anti-corticosterone anti-serum CJM006 protocol, which cross reacts with corticosterone metabolites 100%, and < 10% with other steroids (K. Webster, E. Narayan and N. de Vos, unpublished data). Results were given as FCM concentration (pg g^−1^) on a wet weight basis, and for ease of interpretation were converted to nanogrammes per gramme.

Corticosterone is the primary endogenous adrenal steroid in rodents (Yu et al. 2015). To ensure validation of our FCM extraction via the EIA method, we first demonstrated parallelism between the dilutions of pooled faecal extracts and corticosterone (CJM06 Ab) standard curves. The corticosterone parallelism plot was sufficiently linear. Dilution factors were not required for the samples, based on the > 50% binding point on the corticosterone parallelism curve (Supplementary Fig. S1). We further tested the recovery of exogenous corticosterone added to extracts (85% recovery rate), the sensitivity of the assay (81.26 pg/well), and the degree of intra-assay variation (9.6%) and inter-assay variation (8.6%).

### Behavioural response monitoring

Four behaviours were selected for analyses based on their frequent use as indicators of stress and the ability to measure them in situ minimally invasively through video recordings. Percentage of time spent at the feeder was measured, as it is often the basis of stress and fear studies that, for example, measure giving-up densities, with decreased time spent in foraging activity being indicative of a higher perceived threat level and hence a higher level of stress (Brown, 1988). Percentage of time spent in the nest hide box was measured, as this is a passive retreat low-activity response, with increased time spent in this activity indicative of higher levels of stress (Koolhaas et al. 1999). Distance travelled was measured, as it is an active response that may reflect territoriality, with decreased distances indicative of higher levels of stress and lower activity (Koolhaas et al. 1999). Finally, maximum movement speed was measured (cm/s), with increased speed indicative of higher levels of stress that are frequently paired with reduced activity (Persons and Rypstra 2001).

As these behaviours each reflect responses to stressors in different ways, and to account for our small sample size, we determined that the best way to measure rats’ overall behavioural response to the stressors was to combine measures of the four behaviours into one *activity index*:

$$ \left(\left(\left(\%\mathrm{time}\ \mathrm{at}\ \mathrm{feeder}+\mathrm{distance}\ \mathrm{travelled}\right)\hbox{--} \%\mathrm{time}\ \mathrm{in}\ \mathrm{nest}\ \mathrm{box}\right)\hbox{--} \mathrm{maximum}\ \mathrm{speed}\ \mathrm{travelled}\right) $$

As the activity index is calculated in the same way across the treatments, we did not attempt to standardise it to accommodate the different units of measurement. The biological relevance of the constituent behaviours is thus retained and, given the above logic, a high activity index reflects a more active reaction by rats that can be attributed to a low level of stress, and a low activity index reflects a less active reaction that can be associated with a higher level of stress.

### Statistical analyses

All statistical analyses were performed in R 3.6.1 (R Development Core Team 2019), using generalised linear mixed models (GLMMs) constructed through the *lmer* function in the *lme4* package (Bates et al. 2015). Treatment type was the consistent explanatory fixed effect, but the number of days in captivity was also tested at the time of model selection by comparing the corrected Akaike information criteria of models with and without it included (Burnham and Anderson 2002), using the *AICc* function in the *MuMIn* package (Barton 2019). Models were further refined by assessing random effects through likelihood ratio tests of model reductions using the *ranova* function in the *lmerTest* package (Kuznetsova et al. 2017). To determine the appropriate distribution and link for each model fit—i.e. Gaussian with logarithm link for continuous data—residual plots and Pearson’s dispersion tests were used (Zuur et al. 2009). If a good fit was not observed, or in cases of overdispersion, data were log-transformed and a GLMM with a Gaussian distribution was fitted (Zuur et al. 2009). Wald’s chi-squared tests were used to generate *p* values with the *ANOVA* function in the *car* package (Fox and Weisberg 2018). Post hoc pairwise comparisons between the responses to the domestic cat treatment and all other treatments, and between the 1st and 2nd halves of the night for each treatment independently, were performed using Dunnett’s method for *p* value adjustments through the *emmeans* function in the *emmeans* package (Lenth et al. 2018). Associations between all variables, including the individual behaviour variables that were combined to form the activity index, were assessed using Pearson’s correlations through the *rcorr* function in the *Hmisc* package (Harrell and Dupont 2019). Boxplots were constructed from the raw data using the *ggboxplot* function from the *ggpubr* package (Kassambara 2019).

As moon phase and rainfall could not be controlled, we included their daily measurements for the Macquarie Park area in statistical analyses. Moon phase each night was calculated using an open-source application (vercalendario.info). The data were then converted to a categorical factor with four levels based on the percentage fullness and the shape of the moon: new 0–1% full, crescent 2–49% full, gibbous 50–98% full, and full 99–100% full. Rainfall data were taken from the Bureau of Meteorology (www.bom.gov.au) and converted to a binomial of “yes” or “no” rain across the nocturnal study period. Both the physiological stress and behavioural activity responses considered the same fixed and random effects for the models of best fit. The explanatory fixed effects included in the full models were as follows: treatment (capture, acclimatisation, procedural control, novel herbivore pungent control—koala fur, familiar herbivore pungent control—common brushtail possum fur, predator—domestic cat fur, 1-day post-cat non-treatments, and post-experiment acclimatisation), and number of days in captivity (1–18) (Fig. 1). The random effects included in the full models were as follows: individual identity, moon phase, rainfall, and rat body mass (set as a categorical factor with four levels, based on the general physiology and post-natal development stages by weight class: weanling < 115 g, peri-adolescent 115–250 g, adult 250–400 g, old adult/large male > 400 g) (Fig. 1). As described in the “Materials and methods” section, there are discrepancies between the peak FCM response time for rats, with the common average being 8 h after exposure. Accordingly, to investigate whether the observed behavioural responses were consistent across the full nocturnal study period of 10 h, the activity index was also modelled over the halves of the night that were divided into the first 5 h after sunset and the second 5 h that precedes sunrise. These models included an additional nested fixed effect of night period (1st or 2nd half) by the treatment (as noted above).

## Results

### External influencing factors

All rats were tested negative for toxoplasmosis and thus can be assumed to have an exhibited behaviour clear of any influence of *T*. *gondii*.

The number of days in captivity was not retained in any of the most parsimonious models. Moon phase was negatively correlated with percentage time that rats spent at the feeder (a component of the activity index), with a fuller moon associated with reduced time spent at the feeder (*n* = 80, *r* = − 0.37, *p* < 0.001). Similarly, rainfall was positively correlated with the maximum speed travelled (another component of the activity index); as the amount of rainfall across the night increased so too did the maximum speed travelled (*n* = 80, *r* = 0.27, *p* = 0.01). However, moon phase and rainfall, as random categorical factors, were not retained in the most parsimonious models for the activity index. A correlation matrix considering all variables monitored per night is provided as a supplementary material (Table S1).

### The physiological response to predator odour

**Fig. 2:**
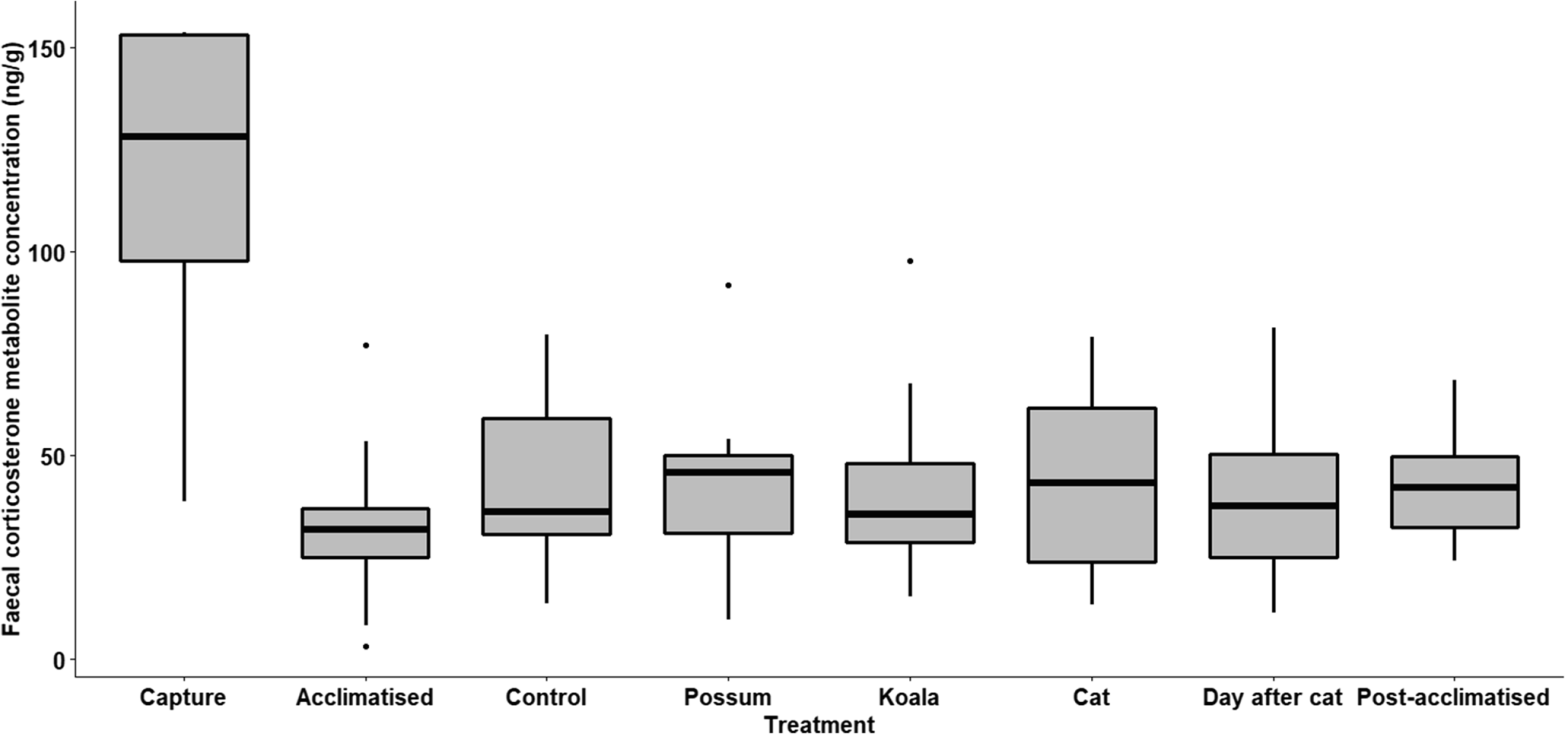
Boxplots based on the raw data for the physiological stress response of faecal glucocorticoid metabolite concentration (FCM ng/g) in wild-captured rats (*Rattus norvegicus* and *R. rattus*) following exposure to an olfactory predator cue from the domestic cat (*Felis catus*), when compared to the olfactory cues of a novel herbivore (koala, *Phascolarctos cinereus*) and a known herbivore (common brushtail possum, *Trichosurus vulpecula*). Observations were also made following acclimatisation (the fifth day after being captured and placed in an enclosure with no treatments), exposure to a procedural control, the day after exposure to the domestic cat treatment (where no treatments were administered), and post-acclimatisation (the fifth day after being exposed to the last treatment). Faecal samples were collected fresh each morning at sunrise, at ~ 12 h after the initial exposure that commenced the night before at sunset

No obvious change in FCM (ng/g) levels to the predator cue (domestic cat fur) was observed in rats (estimated marginal mean FCM under procedural control treatment = 39.1 ng/g ± SE 6.26, compared to estimated marginal mean FCM under cat treatment = 38.1 ng/g ± SE 5.98, *t*_82_ = − 0.14, *p* = 0.887). However, there was a significant increase in FCM in response to capture (estimated marginal mean FCM under procedural control treatment = 39.1 ng/g ± SE 6.26, estimated marginal mean FCM under capture conditions = 98.2 ng/g ± SE 26.07, *t*_82_ = 3.24, *p* = 0.002) (Table 1 and Fig. 2). Based on pairwise assessments of the response of rats to domestic cat odour compared to each treatment, the responses were most similar to the common brushtail possum and koala treatments (Table 1). FCM levels in response to the acclimatisation period were the lowest (Table 1). Individual identity was positively correlated with FCM; as the identity number increased, so too did the FCM level (*n* = 80, *r* = 0.28, *p* = 0.01).

**Table 1:** Analysis of deviance (Wald’s chi-square tests) and post hoc adjusted pairwise comparisons for a model constructed to test the physiological stress response of faecal glucocorticoid metabolite concentration (FCM ng/g) in wild-captured rats (*Rattus norvegicus* and *R. rattus*) following exposure to an olfactory predator cue from the domestic cat (*Felis catus*), compared to the olfactory cues of a novel herbivore (koala, *Phascolarctos cinereus*) and a known herbivore (common brushtail possum, *Trichosurus vulpecula*). Faecal samples were collected fresh each morning at sunrise, at ~ 12 h after the initial exposure that commenced the night before at sunset

Final model: log(FCM ng/g) ~ treatment type + random (animal identity)						
Family: Gaussian		Intercept: procedural control treatment				
Fixed effects		Estimate	SE	d.f.	*T*	*p*
(Intercept)		3.67	0.16	34	22.93	< 2e−16
Capture		0.92	0.28	82	3.24	**0.002**
Acclimatised (5th day)		− 0.39	0.20	82	− 1.88	0.063
Possum		− 0.05	0.24	82	− 0.20	0.842
Koala		− 0.06	0.24	82	− 0.25	0.806
Cat		− 0.03	0.19	82	− 0.14	0.887
Day after cat treatment (no treatment)		− 0.09	0.18	82	− 0.48	0.633
Post-acclimatised (5th day after treatments)		0.05	0.24	82	0.20	0.845
Analysis of deviance table (type III Wald’s chi-square tests)						
Fixed factors				d.f.	*χ*^2^	*p*
(Intercept)				1	526.01	< 2.2e−16
Treatment				7	20.00	**0.006**
Post hoc pairwise comparisons of responses to cat against each treatment, with Dunnett’s *p* value adjustment for 7 tests						
	Contrast	Ratio	SE	d.f.	*t* ratio	*p*
Treatment	Capture/cat	2.58	0.73	82	3.33	**0.008**
	Control/cat	1.03	0.19	82	0.14	0.999
	Acclimatised/cat	0.70	0.14	82	− 1.78	0.338
	Possum/cat	0.98	0.23	82	− 0.09	1.000
	Koala/cat	0.97	0.23	82	− 0.13	1.000
	Day after cat/cat	0.94	0.17	82	− 0.34	0.992
	Post-acclimatised/cat	1.08	0.25	82	0.31	0.994

Significant results (< 0.05) are given in bold text

### The behavioural response to predator odour

**Fig. 3:**
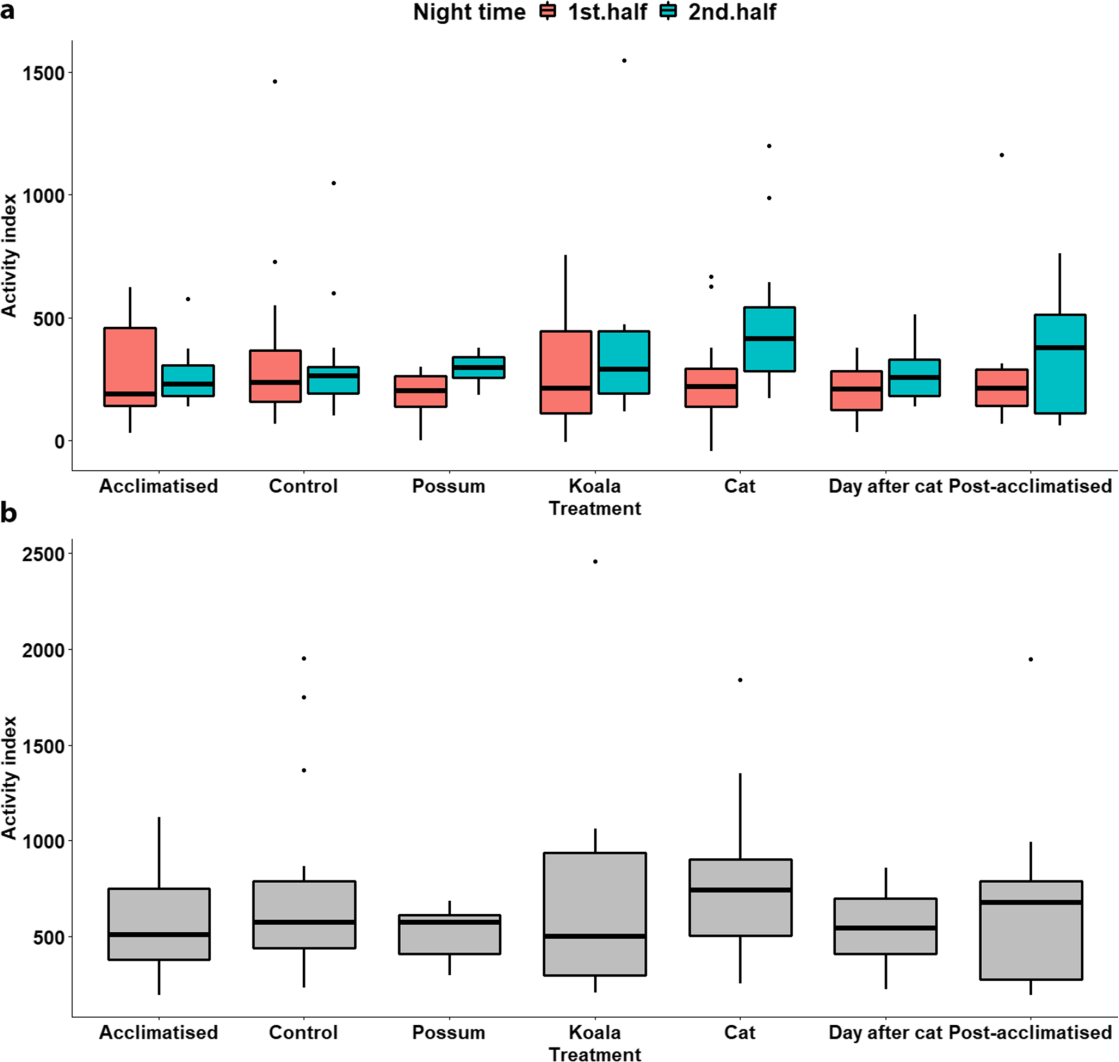
The behavioural response, as measured by the activity index of (((% time at feeder + total distance travelled) − % time in nest box) − maximum speed travelled) for urban wild-captured rats (*Rattus norvegicus* and *R*. *rattus*) following exposure to an olfactory predator cue from the domestic cat (*Felis catus*), compared to the olfactory cues of a novel herbivore (koala, *Phascolarctos cinereus*) and a known herbivore (common brushtail possum, *Trichosurus vulpecula*). Observations were also made following acclimatisation (the fifth day after being captured and placed in an enclosure with no treatments), exposure to a procedural control, the day after exposure to the domestic cat treatment (where no treatments were administered), and post-acclimatisation (the fifth day after being exposed to the last treatment). Behaviour measurements were taken across the first 5 h following sunset (1st) and the 5 h following those—before dawn (2nd) (**a**), and across the full 10-h period (**b**)

No obvious behavioural response indicative of increased levels of stress (low activity index) in response to the predator cue (domestic cat fur) was observed in rats across the full night or halves of the night (estimated marginal mean activity index under the procedural control treatment across the night = 617 ± SE 110.3, compared to that under the cat treatment = 676 ± SE 120.8, *t*_66_ = 0.60, *p* = 0.55; and estimated marginal mean activity index under the procedural control treatment across the halves of the night = 253 ± SE 50.5, compared to that under the cat treatment = 293 ± SE 58.8, *t*_136_ = − 0.76, *p* = 0.447) (Table 2 and Fig. 3). However, the activity index for rats in response to the domestic cat treatment was significantly lower across the first half of the night compared to the second half (estimated marginal mean activity index under the cat treatment for the first half of the night = 209 ± SE 48.6, compared to that for the second half of the night = 411 ± SE 94.2, *t*_136_ = 2.00, *p* = 0.047) (Table 2 and Fig. 3).

**Table 2:** Analysis of deviance (Wald’s chi-square tests) and post hoc adjusted pairwise comparisons for a model constructed to test the behavioural response, activity index: ((( % time at feeder + total distance travelled) − % time in nest box) − maximum speed travelled), for wild-captured rats (*Rattus norvegicus* and *R. rattus*) following exposure to an olfactory predator cue from the domestic cat (*Felis catus*), compared to the olfactory cues of a novel herbivore (koala, *Phascolarctos cinereus*) and a known herbivore (common brushtail possum, *Trichosurus vulpecula*). Observations were taken across the two halves of the nocturnal study period, being the first 5 h after sunset (1st) and the 5 h following those—before dawn (2nd), and across the full survey period of the combined 10 h

Full night final model: log(activity index) ~ treatment type + random (animal identity)											
Family: Gaussian intercept: procedural control treatment											
Halves of the night final model: log(activity index) ~ treatment type + half of the night/treatment + random (animal identity)											
Family: Gaussian intercept: procedural control treatment, 1st half of the night											
Fixed effects		Estimate		SE		d.f.		*t*		*p*	
	Nocturnal survey period	Full	Half	Full	Half	Full	Half	Full	Half	Full	Half
(Intercept)		6.42	5.51	0.18	0.23	14	21	35.93	24.07	5.88^e-15^	< 2^e-16^
Acclimatised (5th day)		− 0.18	− 0.19	0.19	0.28	66	136	− 0.95	− 0.68	0.344	0.501
Possum		− 0.21	− 0.88	0.19	0.28	66	136	− 1.13	− 3.21	0.216	**0.002**
Koala		− 0.10	− 0.03	0.19	0.31	66	136	− 0.53	− 0.10	0.595	0.925
Cat		0.09	− 0.17	0.15	0.23	66	136	0.60	− 0.76	0.550	0.447
Day after cat treatment (no treatment)		− 0.21	− 0.39	0.15	0.22	66	136	− 1.36	− 1.74	0.179	0.084
Post-acclimatised (5th day after treatments)		− 0.11	− 0.15	0.19	0.28	66	136	− 0.57	− 0.53	0.573	0.596
Control: 2nd half of the night		na	0.04	na	0.22	na	136	na	0.17	na	0.869
Acclimatised (5th day): 2nd half of the night		na	0.15	na	0.39	na	136	na	0.38	na	0.702
Possum: 2nd half of the night		na	0.99	na	0.39	na	136	na	2.55	na	**0.012**
Koala: 2nd half of the night		na	0.24	na	0.41	na	136	na	0.57	na	0.568
Cat: 2nd half of the night		na	0.64	na	0.32	na	136	na	2.00	na	**0.047**
Day after cat treatment: 2nd half of the night		na	0.37	na	0.32	na	136	na	1.16	na	0.247
Post-acclimatised: 2nd half of the night		na	0.15	na	0.39	na	136	na	0.38	na	0.702
Analysis of deviance table (type III Wald’s chi-square tests)											
Fixed factors						d.f.		*X*^2^		*p*	
	Nocturnal survey period					Full	Half	Full	Half	Full	Half
(Intercept)						1	1	1291	579.5	< 2e−16	< 2e−16
Treatment						6	6	5.68	12.43	0.460	**0.053**
Treatment: half of the night						na	6	na	9.07	na	0.170
Post hoc pairwise comparisons of responses to cat against each treatment, with Dunnett’s *p* value adjustment for 6 tests											
	Contrast	Ratio		SE		d.f.		*t* ratio		*p*	
	Nocturnal survey period	Full	Half	Full	Half	Full	Half	Full	Half	Full	Half
Treatment by cat treatment	Control/cat	0.91	0.86	0.14	0.14	66	136	− 0.60	− 0.91	0.942	0.821
	Acclimatised/cat	0.76	0.77	0.14	0.15	66	136	− 1.44	− 1.32	0.503	0.581
	Possum/cat	0.74	0.59	0.14	0.12	66	136	− 1.62	− 2.72	0.404	**0.038**
	Koala/cat	0.83	0.94	0.15	0.20	66	136	− 1.02	− 0.28	0.762	0.994
	Day after cat/cat	0.74	0.70	0.11	0.11	66	136	− 1.96	− 2.21	0.226	0.133
	Post-acclimatised/cat	0.82	0.80	0.15	0.16	66	136	− 1.06	− 1.11	0.744	0.709
Post hoc pairwise comparisons for each treatment between the halves of the night											
	Contrast	Ratio		SE		d.f.		*t* ratio		*p*	
Treatment by half of the night	Control: 1st/2nd half	0.96		0.22		136		− 0.16		0.869	
	Acclimatised: 1st/2nd half	0.83		0.26		136		− 0.59		0.559	
	Possum: 1st/2nd half	0.36		0.11		136		− 3.24		**0.001**	
	Koala: 1st/2nd half	0.76		0.26		136		− 0.79		0.430	
	Cat: 1st/2nd half	0.51		0.12		136		− 2.97		**0.003**	
	Day after cat: 1st/2nd half	0.67		0.15		136		− 1.81		0.073	
	Post-acclimatised: 1st/2nd half	0.83		0.26		136		− 0.59		0.559	

Significant results (< 0.05) are given in bold text

Based on pairwise assessments comparing rat responses to domestic cat odour to each treatment over the full night period, the response under the control treatment was the most similar to those observed under the domestic cat treatment (Table 2). The activity index level in response to the common brushtail possum treatment and the day after the cat treatment showed the lowest activity index levels (Table 2). Based on pairwise assessments between the first and second halves of the night for each treatment, the control and both acclimatisation periods showed little change across the halves, and the common brushtail possum and domestic cat treatments had the largest variance in the activity index level across the halves, both of which were significant (*t*_136_ = − 3.24, *p* = 0.001; and *t*_136_ = − 2.97, *p* = 0.003, respectively). Individual identity was negatively correlated with the activity index level; as the identity number increased, the activity index level decreased (*n* = 80, *r* = − 0.55, *p* < 0.001).

There was a significant decrease in the activity index when comparing the response to the possum treatment to that from the control treatment across the halves of the night (estimated marginal mean activity index under procedural control treatment across the halves of the night = 253 ± SE 50.5, compared to that under the possum treatment = 172 ± SE 39.4, *t*_136_ = − 3.21, *p* = 0.002), but not when comparing the data across the full night (Table 2 and Fig. 3). Similar to the response to the domestic cat treatment across the halves of the night, the activity index in response to the common brushtail possum treatment also differed across the first half of the night compared to the second half (estimated marginal mean activity index under the common brushtail possum treatment for the first half of the night = 103 ± SE 28.6, compared to that for the second half of the night = 288 ± SE 80.3, *t*_136_ = 2.55, *p* = 0.012) (Table 2 and Fig. 3).

## Discussion

The physiological and behavioural responses to a stressor that we observed in rats were expressed concordantly as low to no response across each single night duration of exposure. Behavioural activity, however, increased over the second half of each single exposure period to domestic cat and common brushtail possum cues. As such, the results of this pilot study indicate that wild rodent responses to the threat of a predator are nuanced, and while physiological and behavioural responses to the stressor are expressed concordantly they are likely to be modulated, to a degree, by behavioural changes. A form of habituation to a perceived low-level threat may even occur within hours, and for this reason, measurements of both physiological and behavioural responses to a stressor should be taken to effectively capture a wild animal’s stress response. Integumentary scents, such as fur, are more indicative of local predator presence or activity than are urine or scats, as fur has been repeatedly observed to elicit strong and consistent anti-predator responses that include endocrinal and behavioural changes in prey (Blanchard et al. 2003; Blanchard and Blanchard 2004; Masini et al. 2005). As predator fur is recognisable by prey to elicit a response (Banks et al. 2014) and it is energetically more efficient for wild animal responses to the threat of predation by a prevalent predator to be modulated (Johnstone et al. 2012), we consider our interpretation to be the most parsimonious explanation of the low- to no-level responses, and modulation, that we observed.

### Concordant physiological and behavioural responses to a stressor

Both the physiological and behavioural responses we observed varied to some degree among individuals. The levels of FCM increased and the activity index decreased as the identification number of individual increased. This may indicate a concordant response to a stressor that reflects differences in personality, past experience, age, and/or body condition (Bedoya-Pérez et al. 2019). Generally, an animal’s control in handling a stressor has a large effect on its behavioural and physiological coping responses (Dantzer 1989). In conditions where an animal cannot escape from a stressor, as in laboratory experiments, the animal subjected to the stressor generally shows a passive reaction of withdrawal and increased adrenocortical activity (Archer 1979; Henry 1982; Moberg 1985). Despite the animals in our study also being unable to escape, we did not observe obvious increased physiological and reduced activity responses—in fact, it was the opposite and both were concordantly expressed as no physiological response and no significant change in activity. What we observed may be interpreted as wild animals’ coping responses to common low-level stressors. In situ studies of wild animals that show obvious physiological and behavioural responses to a stressor are perhaps then the result of coping responses to stronger perceived stressors, when behaviour changes and activity levels may no longer moderate the stress levels (e.g. ecotourism impacts on hoatzin chicks: Müllner et al. 2004; culling impacts on fallow deer: Pecorella et al. 2016; low-quality habitat effects on wood mice: Navarro-Castilla and Barja 2019).

Containing wild animals can impair their ability to show a measurable physiological response to a stressor due to the chronic stress it may initiate (Dickens et al. 2009). However, the number of days in captivity was not retained in our most parsimonious models, and further, the differences in response by individuals to each successive exposure to each treatment they were exposed to across the 18-day containment period did not show a fluctuation that was consistent with the number of days in captivity. As such, we do not believe that we observed chronic stress due to containment during the treatment period, although such a response was perhaps beginning to take effect around the 18th day (5 days post-treatments) as the FCM was higher then, than in the pre-acclimatisation period.

Similar results to ours, of ostensibly moderate behavioural and/or physiological coping responses to a stressor, have been observed in wild-caught brown and black rats exposed to domestic cat cues (Bramley et al. 2000; Bramley and Waas 2001). The olfactory cues of this predator may be less likely to elicit an obvious coping response in their wild prey, as domestic cat cues are prevalent in urban areas and frequent avoidance responses would be energetically disadvantageous (Lima and Bednekoff 1999). This rationale was applied to the results of a similar study that found no obvious response by black rats to red foxes and domestic dogs in Australia (Carthey and Banks 2018).

### Modulation of response to a stressor

Under the treatments of a known predator, the domestic cat, and a known herbivore and competitor, the common brushtail possum, the activity index was marginally lower in the first half of the night, indicating increased levels of stress-associated behaviours, but as the hours of exposure increased so too did the activity index, indicating reduced stress. Such results suggest a form of habituation or threat downgrading as no further signs of the threat were presented. The disparity in activity index level across the halves of the night was larger under the domestic cat treatment, which may suggest that this cue is perceived by rats as more stressful than cues to competitors. Further, the standard errors were wider under the domestic cat treatment, suggesting variable responses across individual rats. This could reflect previous interactions with domestic cats, or perhaps our small sample size. The response to the common brushtail possum fur may be explained by rats’ antagonised experience with them as a competitor. While common brushtail possums are largely herbivorous, they overlap partly in diet with rats and may be viewed as a competitor (Sweetapple and Nugent 2007). Common brushtail possums are frequently aggressive towards competitors, especially when food resources are scarce (McDonald-Madden et al. 2000). As such, common brushtail possums could exert enough pressure for awareness and aversive responses to them to have developed in the introduced rats.

Our results indicate that stress reactions may be moderated according to the severity of the perceived threat over time, as further information is gathered. Such findings align with previous research showing that wild rodents modulate their behavioural responses to a predator odour cue according to the age or concentration of the cue (e.g. Hegab et al. 2014; Sánchez-Gonzáles et al. 2018), thus indicating that a level of threat processing occurs. Felids similarly alter behaviours that are negatively correlated with cortisol secretion when exposed to a chronic low-level stressor, again indicating an ability to moderate stress impacts through coping behaviours (Carlstead et al. 1993a, b). Laboratory rodents have also been observed to modulate their stress through using grooming as a “displacement” behaviour that has a relaxing effect (Kalueff and Tuohimaa 2004; Smolinsky et al. 2009). Our study was limited by a single regulated stressor treatment per night, but increasing the threat level incrementally across the night by using visual and audio or other cues would give deeper insight into the stages and limitations of modulated responses. Indeed, a recent study on wild-caught brown rats found that multiple predation cues, as combinations of both direct and indirect cues, can interact to amplify the behavioural response to the stressor (Farnworth et al. 2020).

### Comparison to laboratory-raised rodent findings

When compared to previous findings on laboratory-based brown rats that displayed obvious physiological and behavioural stress through increased plasma corticosterone levels and reduced activity in response to domestic cat presence (Blanchard et al. 1998), our results show a much more muted response. Although this difference may not be due solely to the laboratory rat—wild-caught rat dichotomy, the presence of a predator is likely to invoke a higher-level stress response than the cue of that predator (Bedoya-Pérez et al. 2019). Our results also differ from other laboratory-based studies of brown rat responses to domestic cat cues, which have found obvious behavioural (hiding) and physiological stress responses (increased plasma corticosterone levels) (File et al. 1993). However, both File et al. (1993) and Blanchard et al. (1998) reported similar results to ours in that each observed a form of habituation to domestic cat presence or cues. The overnight threat downgrading and partial habituation we detected may therefore provide further insight into how wild rats cope with domestic cat presence.

### Prospective conservation applications

As our results describe the responses of brown and black rats to the stressor of an olfactory cue from a predator that they co-evolved with and to two herbivores that they did not co-evolve with, they may also provide insight into how native rodents could respond to the introduced domestic cat. Native Australian rodents have been in contact with cats for the last 200 years and, like small mammals elsewhere in the world, face significant threats from domestic cat predation (e.g. Dickman 2009; Woinarski et al. 2015; Flockhart et al. 2016; Kikillus et al. 2017; Legge et al. 2017). This vulnerability likely arises when prey are naïve to the threat that domestic cats present (Banks and Dickman 2007; Banks et al. 2018). Our results show that brown and black rats, which co-evolved with domestic cats, modulate responses to domestic cat odour within a single night. Similarly, they also appear to modulate their responses within a single night when exposed to the odour of a frequently encountered native competitor, the common brushtail possum, with which they have not co-evolved. This being the case, perhaps Australian native rodents are able to respond similarly and form a modulated response to the odour of their introduced predator. Repeating our study on native rodents, especially those that persist in urban environments (Ives et al. 2016), will help to inform debates about naiveté responses. Better understanding of how small prey animals respond to stressors created by the presence of predators should allow for more targeted wildlife management. For example, if behaviour is used to mitigate physiological responses to a stressor, this may be facilitated by ensuring the retention of habitat of sufficient complexity that allows coping behaviours to be expressed.

## Conclusion

We conclude that physiological and behavioural responses to a stressor are expressed concordantly, at least when the threat is perceived as low, but can be modulated over time by behavioural activity changes. If taken on their own, they may not adequately show the nuanced response to stressors that may occur in the wild, or the energetic costs that may be associated with modulating the stress response. Responses to stressors in wild-caught animals appear to vary from those of laboratory-raised animals, the former being shaped by evolutionary history and prior encounters with stressors, and the latter constrained by conditions that often preclude the expression of modulating behaviours. Our findings support arguments that predictions about wildlife responses to stressors should not be based on observations of laboratory-raised animal responses, and that in situ measurements of behavioural responses should be taken concurrently with those of physiological responses to a stressor. This should allow robust results to be gained, helping to fill knowledge gaps about stress responses by wildlife in their natural habitats and how to manage them.

## Supplementary information

ESM 1 (DOCX 20 kb)

## Data Availability

Data from the current study are available from the corresponding author upon reasonable request.
