# Supplementary material for: Are physiological and behavioural responses to stressors displayed concordantly by wild urban rodents?
Source: Naturwissenschaften. 2021 Jan 7;108(1):5. doi: 10.1007/s00114-020-01716-8 (PMC7790802; doi:10.1007/s00114-020-01716-8)
Supplement: Supplementary file 1 — (DOCX 20 kb) [file 114_2020_1716_MOESM1_ESM.docx]

**Are physiological and behavioural responses to stressors displayed concordantly by wild urban rodents?**

Loren L. Fardell^1*^, Miguel A. Bedoya-Pérez^1,2,3^, Christopher R. Dickman^1^, Mathew S. Crowther^1^, Chris R. Pavey ^4^, Edward J. Narayan ^5^

^1^School of Life and Environmental Sciences, The University of Sydney, New South Wales, 2006 Australia

^2^School of Psychology, The University of Sydney, New South Wales, 2006 Australia

^3^Brain and Mind Centre, The University of Sydney, New South Wales, 2006 Australia

^4^CSIRO, Land and Water, PMB 44 Winnellie, 0822, Northern Territory, Australia

^5^School of Agriculture and Food Sciences, The University of Queensland, Queensland, Australia

**^*^Corresponding author:** School of Life and Environmental Sciences, The University of Sydney, New South Wales, 2006 Australia. Email: loren.fardell@gmail.com

**Supplementary information**

**Supplementary Fig. S1:** Parallelism curve between the dilutions of pooled faecal extracts and a corticosterone (CJM06 Ab) standard curve, to validate the faecal glucocorticoid metabolite extraction via the enzyme immuno assay method used to determine brown and black rat (*Rattus norvegicus, R. rattus*) physiological stress responses to an olfactory predator cue – domestic cat (*Felis catus*) fur. The corticosterone parallelism provided linear displacement curve. Based on the >50% binding point on the parallelism curve, no dilution factors were required for any of the samples.

**Supplementary Table S1:** Pairwise Pearson correlation coefficient, r values given across a scale where a value of 1 indicates a strong positive correlation, a value of 0 indicates no correlation, and a value of -1 indicates strong negative correlation. The according p-values are given in brackets (*n* = 80). During the surveys periods, the rain and moon phases were not able to be controlled for, hence they have been included in the statistical analyses. There are correlations listed for these factors that are interpretable based on the days, and that different groups of individuals, as well as based on natural rationale.

|  | Day | Animal identity | Treatment | FCM (ng/g) | Weight | Moon phase | Rain | Distance travelled | Feeder time | Nest time | Max speed | Activity index |
| --- | --- | --- | --- | --- | --- | --- | --- | --- | --- | --- | --- | --- |
| Day | 1 | 0 (1.00) | 0.21 (0.06) | 0.06 (0.58) | 0 (1.00) | **-0.25 (0.02)** | 0.07 (0.52) | -0.03 (0.77) | 0 (0.99) | 0.09 (0.44) | 0.01 (0.95) | -0.04 (0.75) |
| Animal identity | 0 (1.00) | 1 | 0 (1.00) | **0.28 (0.01)** | 0.13 (0.25) | **0.38 (<0.001)** | -0.16 (0.16) | **-0.55 (<0.001)** | -0.02 (0.84) | 0.19 (0.09) | -0.13 (0.25) | **-0.55 (<0.001)** |
| Treatment | 0.21 (0.06) | 0 (1.00) | 1 | -0.07 (0.53) | 0 (1.00) | -0.06 (0.63) | 0.1 (0.39) | -0.1 (0.37) | **0.29 (0.009)** | -0.01 (0.96) | 0.05 (0.66) | -0.1 (0.38) |
| FCM (ng/g) | 0.06 (0.58) | **0.28 (0.01)** | -0.07 (0.53) | 1 | -0.2 (0.07) | 0.16 (0.16) | 0.1 (0.38) | -0.21 (0.06) | 0.11 (0.35) | 0.18 (0.10) | -0.09 (0.43) | -0.21 (0.06) |
| Weight | 0 (1.00) | 0.13 (0.25) | 0 (1.00) | -0.2 (0.07) | 1 | -0.04 (0.71) | 0.02 (0.88) | -0.05 (0.67) | -0.14 (0.20) | 0.01 (0.93) | 0.07 (0.52) | -0.05 (0.66) |
| Moon phase | **-0.25** (**0.02**) | **0.38** (**<0.001**) | -0.06 (0.63) | 0.16 (0.16) | -0.04 (0.71) | 1 | **-0.32 (0.003)** | -0.04 (0.73) | **-0.37 (<0.001)** | -0.21 (0.07) | -0.15 (0.19) | -0.03 (0.77) |
| Rain | 0.07 (0.52) | -0.16 (0.16) | 0.1 (0.39) | 0.1 (0.38) | 0.02 (0.88) | **-0.32 (0.003)** | 1 | -0.05 (0.65) | 0.19 (0.09) | 0.18 (0.12) | **0.27 (0.02)** | -0.06 (0.61) |
| Distance travelled | -0.03 (0.77) | **-0.55 (<0.001)** | -0.1 (0.37) | -0.21 (0.06) | -0.05 (0.67) | -0.04 (0.73) | -0.05 (0.65) | 1 | -0.14 (0.21) | **-0.29 (0.01)** | 0.14 (0.22) | **1 (<0.001)** |
| Feeder time | 0 (0.99) | -0.02 (0.85) | **0.29 (0.009)** | 0.11 (0.35) | -0.14 (0.20) | **-0.37 (<0.001)** | 0.19 (0.08) | -0.14 (0.21) | 1 | **0.27 (0.01)** | -0.18 (0.11) | -0.14 (0.21) |
| Nest time | 0.09 (0.44) | 0.19 (0.09) | -0.01 (0.96) | 0.18 (0.10) | 0.01 (0.93) | -0.21 (0.06) | 0.18 (0.12) | **-0.29 (0.009)** | **0.27 (0.01)** | 1 | **-0.26 (0.02)** | **-0.31 (0.005)** |
| Max speed | 0.01 (0.95) | -0.13 (0.25) | 0.05 (0.66) | -0.09 (0.43) | 0.07 (0.52) | -0.15 (0.19) | **0.27 (0.01)** | 0.14 (0.25) | -0.18 (0.11) | **-0.26 (0.02)** | 1 | 0.13 (0.24) |
| Activity index | -0.04 (0.75) | **-0.55 (<0.001)** | -0.1 (0.38) | -0.21 (0.06) | -0.05 (0.66) | -0.03 (0.77) | -0.06 (0.61) | **1 (<0.001)** | -0.14 (0.21) | **-0.31 (0.005)** | 0.13 (0.24) | 1 |
